# Protection against APOE4-associated aging phenotypes with a longevity-promoting intervention

**DOI:** 10.1093/geroni/igab046.3427

**Published:** 2021-12-17

**Authors:** Cassandra McGill, Amy Christensen, Wenjie Qian, Caleb Finch, Berenice Benayoun, Christian Pike

**Affiliations:** 1 University of Southern California, Gardena, California, United States; 2 University of Southern California, University of Southern California, California, United States; 3 University of Southern California, Los Angeles, California, United States

## Abstract

Two of the primary risk factors for late onset Alzheimer’s Disease (AD) are aging and APOE genotype. While the causal relationship between aging and AD is not well defined, there are strong leads from shared phenotypes such as decreased metabolic function and increased inflammation. APOE genotype may be linked to AD phenotypes through the regulation of aging processes. The NIA Interventions Testing Program (ITP) recently found that 17α-estradiol (17αE2) treatment increases rodent lifespan. Since 17αE2 acts upon systemic and neural pathways associated with AD pathology, we propose that 17αE2 may be a pleiotropic intervention strategy. Further, because APOE4 is associated with a senescent phenotype, 17αE2 may have APOE genotype-specific effects. Using 10-month-old APOE3 or APOE4 targeted replacement male mice maintained on normal chow with and without 14.4 ppm 17aE2 for 20 weeks, our initial results indicate genotype differences in the efficacy of 17αE2 across multiple outcomes. APOE4 mice exhibited an aged phenotype compared to APOE3, with APOE4 mice having a higher frailty index; however, 17αE2 treatment reduced the frailty index most strongly in APOE4 mice. APOE4 mice were impaired across multiple metabolic measures including body weight, plasma leptin, and hepatic steatosis. 17αE2 significantly attenuated the APOE4 metabolic phenotype. These data confirm and extend prior findings that APOE4 is linked to progeroid effects both peripheral and neural outcomes associated with AD risk. Importantly, 17αE2 significantly improved a range of measures, but showed the strongest effects in the APOE4 genotype. This research was funded by the Cure Alzheimer's Fund.

